# Prediction of crude protein digestibility of animal by-product meals for dogs by the protein solubility in pepsin method*

**DOI:** 10.1017/jns.2014.32

**Published:** 2014-09-30

**Authors:** Iris M. Kawauchi, Nilva K. Sakomura, Cristiana F. F. Pontieri, Aline Rebelato, Thaila C. Putarov, Euclides B. Malheiros, Márcia de O. S. Gomes, Carlos Castrillo, Aulus C. Carciofi

**Affiliations:** 1 Grandfood Indústria e Comércio Ltda, Dourado, SP, Brazil; 2 College of Agrarian and Veterinarian Sciences (FCAV), São Paulo State University (UNESP), Via de Acesso Professor Paulo Donato Castellane, Jaboticabal 14.884-900, SP, Brazil; 3 School of Veterinary Medicine and Animal Science (FMVZ), Sao Paulo State University (UNESP), Distrito de Rubião Junior, Botucatu 18.618-970, SP, Brazil; 4 Camilo Castelo Branco University (UNICASTELO), Av. Hilário da Silva Passos, Descalvado 13690-000, SP, Brazil; 5 Animal Production and Food Sciences Department, University of Zaragoza, Miguel Servet, 177, Zaragoza 50.013, Spain

**Keywords:** Dog food, *In vitro* methods, Meat and bone meal, Poultry by-product meal, CP, crude protein, MBM, meat and bone meal, PM, poultry by-product meal, PSP, protein solubility in pepsin

## Abstract

Animal by-product meals have large variability in crude protein (CP) content and digestibility. *In vivo* digestibility procedures are precise but laborious, and *in vitro* methods could be an alternative to evaluate and classify these ingredients. The present study reports prediction equations to estimate the CP digestibility of meat and bone meal (MBM) and poultry by-product meal (PM) using the protein solubility in pepsin method (PSP). Total tract CP digestibility of eight MBM and eight PM samples was determined in dogs by the substitution method. A basal diet was formulated for dog maintenance, and sixteen diets were produced by mixing 70 % of the basal diet and 30 % of each tested meal. Six dogs per diet were used to determine ingredient digestibility. In addition, PSP of the MBM and PM samples was determined using three pepsin concentrations: 0·02, 0·002 and 0·0002 %. The CP content of MBM and PM ranged from 39 to 46 % and 57 to 69 %, respectively, and their mean CP digestibility by dogs was 76 (2·4) and 85 (2·6) %, respectively. The pepsin concentration with higher Pearson correlation coefficients with the *in vivo* results were 0·0002 % for MBM (*r* 0·380; *P* = 0·008) and 0·02 % for PM (*r* 0·482; *P* = 0·005). The relationship between the *in vivo* and *in vitro* results was better explained by the following equations: CP digestibility of MBM = 61·7 + 0·2644 × PSP at 0·0002 % (*P* = 0·008; *R*^2^ 0·126); and CP digestibility of PM = 54·1 + 0·3833 × PSP at 0·02 % (*P* = 0·005; *R*^2^ 0·216). Although significant, the coefficients of determination were low, indicating that the models were weak and need to be used with caution.

Animal by-product meals have been (and continue to be) the primary ingredient responsible for the growth and expansion of the global pet food industry because they provide most of the protein, fat and minerals of the diets^(^^1^^)^. However, one difficulty in the use of these ingredients is that they have highly variable chemical composition and quality.

Manufacturers of animal by-products use different criteria to select and determine which raw materials can be directly used for human or animal consumption, are further processed^(^^2^^)^, or are disposed off. In addition, processing conditions may be markedly different between suppliers, leading to the production of meals of different quality^(^^3^^)^. It is recommended^(^^4^^)^ that hair, feathers, hooves, horns, blood and other residues should be present in animal by-products only in the minimal amounts that occur naturally. However, there are several methods of waste separation, which complicates efforts to standardise by-products. In addition, the time between ingredient production in the slaughterhouse and final rendering may differ among processing conditions, leading to variable reductions in the nutrient quality after microbial degradation.

All of these factors indicate the importance for pet food companies to have standard quality criteria for receiving ingredients. These criteria must include chemical and microbiological tests to help to define the nutritional value and safety of the ingredients before their receipt by companies. One major limitation, however, is that these evaluations do not provide information about ingredient digestibility by dogs. *In vivo* methods are the standard way to determine ingredient quality, allowing determinations of digestibility and palatability, among others. However, they require a specific infrastructure, time and expensive animal care that increase costs. Moreover, there is increasing concern about animal welfare in experimental settings^(^^5^^)^. In addition, *in vivo* tests are time consuming, lasting several weeks, and are not suitable for use during ingredient receipt. *In vitro* evaluation, on the other hand, is a practical and feasible alternative to obtain data more rapidly and relatively inexpensively.

For quality control, animal by-product meals are usually evaluated by pet food companies through the protein solubility in pepsin (PSP) method. The concentration of pepsin that best represents the *in vivo* digestion of dogs, however, has not yet been established. The standardisation of this technique will be important to better define the protein quality of ingredients used in pet food, but the validation of *in vitro* methods must be based on the degree of relationship between the *in vivo* and *in vitro* results^(^^6^^)^. Considering this need, the present study reports prediction equations to estimate the crude protein (CP) digestibility of meat and bone meal (MBM) and poultry by-product meal (PM) for dogs, using the PSP method.

## Materials and methods

The study was conducted at the Laboratory of Research on Nutrition and Nutritional Diseases of Dogs and Cats, UNESP, Jaboticabal, Brazil. All of the procedures were approved by the Ethics and Animal Welfare Committee of the College of Agrarian and Veterinarian Sciences, Sao Paulo State University according to the Brazilian animal protection law (protocol no. 027570/10). Samples of eight MBM and eight PM produced in different animal by-products industries located in South-eastern and Southern Brazil were selected. All of the samples were marketed for the pet food industries and were evaluated by *in vivo* (digestibility trials with dogs) and *in vitro* (PSP) methods.

### Digestibility experiment

Eighteen healthy adult beagle dogs with an average age of 5·7 years old (sem 0·534) and 12·01 kg body weight (sem 0·340) were used in digestibility trials. The digestibility trials were performed to assess the CP digestibility of the MBM and PM samples using the substitution method^(^^7^^–^^9^^)^. For each protein source (MBM or PM) the dogs were distributed in a randomised block design with nine diets in total (a control diet plus eight diets composed of each of the protein source samples in the study), two dogs per diet in each block and three blocks of eighteen animals each for a total of six replicates per diet.

A control diet was formulated for dog maintenance^(^^4^^)^ (Table 1). Sixteen diets, eight using MBM and eight using PM, were obtained by mixing 70 % of the control diet with 30 % of each animal by-product meal in the study (on as-fed basis). The final CP concentration of the diets using MBM ranged from 29·2 to 31·6 %, and the final CP concentration of diets using PM ranged from 33·4 to 37·9 %, both on an as fed-basis.

**Table 1.** Ingredient and chemical compositions (as-fed basis) of the control diet

Ingredient composition	%
Maize	38·23
Broken rice	18·00
Soyabean meal	17·23
Maize gluten meal	9·21
Poultry fat	7·15
Poultry by-product meal	5·00
Minor ingredients*	5·18
Total	100
Chemical composition	%, as-fed basis
DM	89·86
Crude protein	23·78
Acid-hydrolysed fat	12·28
Gross energy (MJ/kg of diet)	18·47

*Dicalcium phosphate, calcium carbonate, potassium chloride, sodium chloride, choline chloride, methionine, lysine, vitamin and mineral supplement, palatability enhancer, mould inhibitor and antioxidant.

The final diets (mixture of the control diet and the protein source) were mixed and ground in a hammer mill (Model 4, D'Andrea) fitted with a 0·8 mm screen before being extruded and kibbled under identical processing conditions in a single-screw extruder (Mab 400S, Extrucenter) in the extrusion facility of the College of Agrarian and Veterinarian Sciences, São Paulo State University. The manufacturing process was controlled by adjusting the kibble density to between 390 and 455 g/l (on as-fed basis) every 20 min to ensure consistent cooking and kibble quality (size and expansion). The extruder pre-conditioning temperature was kept above 90°C. Water, steam, screw speed and ration flux were adjusted according to each diet, and the extrusion temperature varied between 107 and 119°C.

During the digestibility trial, the dogs were kept in the individual stainless steel metabolic cages (100 × 100 × 100 cm^3^) equipped with a system to separate faeces and urine for collection. The dogs were fed individually calculated amounts of food. To this end, the food metabolisable energy content was calculated from their chemical compositions (using the equation proposed by the NRC^(^^10^^)^ that consider the crude fibre effect on energy digestibility), and the dogs were fed 130 kcal/kg^0·75^ per d^(^^10^^)^. Water was available *ad libitum*. The daily food amount was divided equally between meals, placed at 08·00 and 16·00 h, and the dogs were allowed to eat for 30 min. Any remaining food was collected, and the intake was recorded. Each digestibility evaluation latest 10 d, using the first five for diet adaptation and the last 5 d for total collection of faeces^(^^4^^)^. On the first day of collection (day 6), all of the faeces were removed from the cages and discarded before 07·30 h, and the total faecal output for each dog was collected from this point onward for the next 5 d. Faeces were collected twice per day and pooled by dog. The faecal samples were weighed and frozen (−15°C) until the laboratory analyses.

Before chemical analysis, the faecal samples were thawed and dried in a forced air oven (320-SE, Fanem) at 55°C for 72 h. Dried faecal samples and diets were ground in a cutting mill (Mod MA-350) fitted with a 1 mm screen. The MBM and PM samples in the study, the seventeen diets and all of the faecal samples were analysed for DM and CP by standard methods^(^^11^^)^. All of the analyses were carried out in duplicate and repeated when the CV was greater than 5 %.

The coefficient of total tract apparent digestibility of the CP in the control and experimental diets was calculated according to the quantitative collection of faeces method^(^^4^^)^. The *in vivo* CP digestibilities of the MBM and PM samples were calculated based on the CP digestibility values of the control diet, the CP digestibility values of the experimental diets (the diets composed of 70 % control diet and 30 % protein source in evaluation), and the inclusion level of the MBM or PM, corrected for the DM-basis^(^^7^^)^:

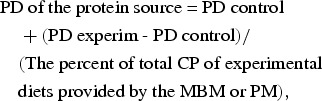

where PD of the protein source is the coefficient of the total tract apparent digestibility of the CP of the MBM or PM samples, PD control is the coefficient of the total tract apparent digestibility of the CP of the control diet and PD experiment is the coefficient of the total tract apparent digestibility of the CP in the diets composed of a mixture of the control diet and the protein source under study.

### Protein solubility by the pepsin method

The PSP of the eight MBM and eight PM samples were determined at three pepsin concentrations: 0·02, 0·002 and 0·0002 %. The analytical procedure followed the method described by Association of Official Analytical Chemists^(^^11^^)^, except for the recommended pepsin concentration. The ground MBM and PM samples were washed with petroleum diethyl ether to remove the fat from the samples and were incubated in an incubator shaker (Innova 44 stackable shaker. Eppendorf) at 45°C and 50 rpm for 16 h with the different pepsin solutions (P7000, Sigma-Aldrich). The insolubilised residue was filtered using filter papers (Whatman ref. 10300211, pore size <2 µm, VWR International eurolab), dried, and its nitrogen content was determined. The PSP was calculated according to the procedure described by the Association of Official Analytical Chemists^(^^11^^)^.

### Statistical analysis

The Pearson correlation coefficients between the *in vivo* CP digestibility results determined in the digestibility trials with dogs and the PSP values obtained *in vitro* with the three pepsin concentrations were determined. The pepsin concentration that correlated better with the *in vivo* results was used to develop the prediction equations for the MBM and PM CP digestibility. To establish the prediction equations, simple linear regressions were performed that considered the PSP results as an independent variable and the *in vivo* digestibility values as a dependent variable. Values of *P* < 0·05 were considered significant. The SAS software version 9·0 (SAS Institute, Cary, NC, USA) was used for the data analysis.

## Results

The CP concentrations of the studied MBM and PM samples ranged from 39·8 to 46·6 % and 57·8 to 69·1 %, respectively (Table 2). The *in vivo* digestibility coefficients of the CP of the MBM samples ranged from 72·6 to 79·4 %, and the pepsin concentration which better correlated with the *in vivo* results was 0·0002 % (*r* 0·380; *P* = 0·008). The prediction equation that represented this relationship was as follows: CP digestibility of MBM = 61·7 + 0·2644 × PSP at 0·0002 % (*P* = 0·008; *R*^2^ 0·126). The *in vivo* CP digestibility of the studied PM samples ranged from 80·4 to 89·1 %, and the pepsin concentration that correlated best with the *in vivo* results was 0·02 % (*r* 0·482; *P* = 0·005). This relationship was expressed by the following equation: CP digestibility of PM = 54·1 + 0·3833 × PSP at 0·02 % (*P* = 0·005; *R*^2^ 0·216).

**Table 2.** Crude protein (CP) concentration (%, as-fed basis), *in vivo* CP digestibility for dogs calculated by the substitution method* and *in vitro* protein solubility in three pepsin concentrations of the meat and bone meal and poultry by-product meal samples under study

Animal by-product meal	CP concentration (%)	*In vivo* CP digestibility(%)	CP solubility in pepsin at 0·02%	CP solubility in pepsin at 0·002%	CP solubility in pepsin at 0·0002%

*Meat and bone meal*					
A	41·0	74·0	79·4	60·7	41·6
B	39·8	76·1	87·5	74·6	53·4
C	46·1	75·1	82·2	71·3	55·1
D	44·9	77·3	85·3	69·4	54·0
E	46·6	75·4	87·5	70·0	57·6
F	43·2	78·9	89·2	77·3	62·2
G	46·5	79·4	86·4	81·8	61·4
H	41·1	72·6	93·8	79·1	52·0
Mean	43·6	76·1	86·4	73·0	54·7
sd	2·75	2·35	4·14	6·31	6·09
Pearson correlation†	–	–	−0·006	0·218	0·380
*P* value‡	–	–	0·969	0·137	0·008
*Poultry by-product meal*					
A	68·7	86·0	82·8	68·0	52·1
B	59·8	82·6	71·0	60·8	42·1
C	69·1	89·1	83·5	72·5	61·6
D	62·5	83·7	79·9	66·3	54·4
E	60·9	86·1	85·1	67·0	54·5
F	57·8	80·4	76·5	70·6	51·3
G	61·0	84·8	81·8	65·1	51·9
H	66·9	84·0	74·7	67·2	51·6
Mean	63·3	84·6	79·4	67·5	52·4
sd	4·32	2·63	4·64	3·47	5·02
Pearson correlation†	–	–	0·482	0·288	0·470
*P* value‡	–	–	0·005	0·047	0·007

* Calculated with six dogs per diet^(^^7^^)^.

†Pearson correlation coefficient between the *in vivo* crude protein (CP) digestibility results determined in the digestibility trials with dogs and the pepsin solubility of CP values obtained *in vitro* with the three pepsin concentrations^(^^11^^)^.

‡*P* value of the Pearson correlation.

## Discussion

The MBM samples evaluated presented low CP contents compared with values described in the scientific literature^(^^3^^,^^10^^,^^12^^–^^15^^)^. However, when compared with the values reported on Brazilian feed composition tables^(^^16^^)^ and local studies^(^^17^^)^, it is possible to observe that the low CP concentration of MBM is an intrinsic characteristic of the raw material used to produce this ingredient in Brazil. The MBM samples presented high ash content (38·7 % on as-fed basis; data not shown), suggesting large inclusion of bones. The CP contents of the PM samples evaluated were slightly lower than the contents recorded by Dozier *et al.*^(^^18^^)^, who evaluated ten PM classified as pet food grade. Those authors collected samples of PM produced in industries located in Alabama, Delaware, Georgia, North Carolina, Tennessee and Virginia and found that the CP contents ranged from 63·0 to 69·3 % (mean of 66·1 %). In addition, in the present study, a high variation in CP concentrations of PM produced in Brazil compared with the values reported by authors in the USA was noted (sd = 4·32 *v.* 1·90, respectively). It has been suggested that a lower standard applies to the PM classified as pet food grade in Brazil and that pet food companies in Brazil may need to pay greater attention when acquiring this ingredient. However, despite the variations in the CP contents of the PM, high CP digestibilities were observed, indicating that most of the CP in the ingredient originated from meat components and not from nitrogen-rich components of low nutritional value, such as feathers.

Greater differences between the PSP results of the evaluated samples were observed when lower concentrations of pepsin were used for both MBM and PM. In addition, when the enzyme concentration was reduced, the protein solubilities were also reduced and numerically distanced from the *in vivo* data. For MBM, however, it was observed that only the pepsin percentage of 0·0002 % provided PSP values that showed significant correlation with the *in vivo* results, suggesting that when higher concentrations of the enzyme were used, the *in vitro* values were too high and did not adequately discriminate between the high and low digestibility values observed in dogs. This finding may be justified by the fact that insoluble and less digestible proteins become soluble at high concentrations of pepsin, and consequently, the solubility results do not match with values determined *in vivo* and have low biological significance.

It also became clear that, as was already known, the PSP is a qualitative and not a quantitative method, which may allow for discrimination between more or less soluble samples of MBM and PM. To better use the method during the *in vitro* evaluation and classification of animal by-product meals it was necessary, however, to generate an equation to translate the results for solubility in pepsin into terms of dog digestibility, as already available for other species^(^^19^^,^^20^^)^. However, the prediction equations proposed in the present study, even though statistically significant, presented low determination coefficients and need to be used with caution. It is possible that the small number of samples exerted some influence on this low adjustment as well as the reduced variation observed for the *in vivo* values, which were relatively close between samples. Furthermore, it is important to note that there are many sources of variation in studies involving *in vitro* methodologies, including the source of the evaluated ingredient, particular variations related to the different enzymes marketed, the enzyme concentration and purity, and the range of the pH during the assessment, among others.

Unfortunately, no other prediction equations to estimate MBM and PM for extruded dog foods based on the PSP method are available to compare with the present findings. Therefore, the data presented are an important starting point for the use and application of the PSP method as a tool to evaluate the nutritional quality of MBM and PM, and this method deserves further study.
